# Patient Satisfaction With Telemedicine During the COVID-19 Pandemic: Retrospective Cohort Study

**DOI:** 10.2196/20786

**Published:** 2020-09-09

**Authors:** Ashwin Ramaswamy, Miko Yu, Siri Drangsholt, Eric Ng, Patrick J Culligan, Peter N Schlegel, Jim C Hu

**Affiliations:** 1 Department of Urology Weill Cornell Medicine New York, NY United States

**Keywords:** telemedicine, medicine, pandemics, patient satisfaction, remote consultation, disruptive technology, medical informatics, health care delivery, practice patterns, physicians, health policy, health services research, health care reform, COVID-19

## Abstract

**Background:**

New York City was the international epicenter of the COVID-19 pandemic. Health care providers responded by rapidly transitioning from in-person to video consultations. Telemedicine (ie, video visits) is a potentially disruptive innovation; however, little is known about patient satisfaction with this emerging alternative to the traditional clinical encounter.

**Objective:**

This study aimed to determine if patient satisfaction differs between video and in-person visits.

**Methods:**

In this retrospective observational cohort study, we analyzed 38,609 Press Ganey patient satisfaction survey outcomes from clinic encounters (620 video visits vs 37,989 in-person visits) at a single-institution, urban, quaternary academic medical center in New York City for patients aged 18 years, from April 1, 2019, to March 31, 2020. Time was categorized as pre–COVID-19 and COVID-19 (before vs after March 4, 2020). Wilcoxon-Mann-Whitney tests and multivariable linear regression were used for hypothesis testing and statistical modeling, respectively.

**Results:**

We experienced an 8729% increase in video visit utilization during the COVID-19 pandemic compared to the same period last year. Video visit Press Ganey scores were significantly higher than in-person visits (94.9% vs 92.5%; *P*<.001). In adjusted analyses, video visits (parameter estimate [PE] 2.18; 95% CI 1.20-3.16) and the COVID-19 period (PE 0.55; 95% CI 0.04-1.06) were associated with higher patient satisfaction. Younger age (PE –2.05; 95% CI –2.66 to –1.22), female gender (PE –0.73; 95% CI –0.96 to –0.50), and new visit type (PE –0.75; 95% CI –1.00 to –0.49) were associated with lower patient satisfaction.

**Conclusions:**

Patient satisfaction with video visits is high and is not a barrier toward a paradigm shift away from traditional in-person clinic visits. Future research comparing other clinic visit quality indicators is needed to guide and implement the widespread adoption of telemedicine.

## Introduction

New York City was the world’s COVID-19 epicenter in early 2020 [1]. As of May 8, 2020, the five boroughs had 175,997 confirmed cases and 14,381 deaths, comprising 14% of all confirmed cases and 19% of all deaths from COVID-19 in the United States [2]. Health care providers postponed elective surgeries, expanded intensive care unit (ICU) capacity, deployed nursing and physician staff, and rapidly transitioned most clinic encounters to telemedicine (defined here as synchronous video visits) [3].

Historically, telemedicine focused on rural medicine [4] and/or moved forward incrementally through institutional initiatives [5]. The widespread adoption of telemedicine associated with the COVID-19 pandemic was unprecedented and may have a significant and durable impact on health care delivery. Telemedicine has not commonly been tested in disaster settings [6]. It was an essential component of the medical response to COVID-19 by reducing demand on strained health care infrastructure and enabling health care needs to be met at home while reducing exposure for patients and medical staff [7,8]. Patient demand for telemedicine outstripped the ability of health care providers to supply it [9]. In early March, the Centers for Medicare and Medicaid Services established telemedicine payment parity with in-person visits, suspended licensure and malpractice insurance restrictions, and waived HIPAA (Health Insurance Portability and Accountability Act) regulations regarding video visits [10] to limit barriers to widespread adoption of telemedicine.

We examined patient acceptance of video visits by comparing Press Ganey patient satisfaction scores for video vs in-person visits at an urban, quaternary referral, academic medical center from April 1, 2019, to March 31, 2020. We hypothesized that there would be no difference in Press Ganey patient satisfaction scores between video and in-person visits. We captured one month of clinic visits during the COVID-19 pandemic and sought to determine the factors associated with patient satisfaction during this time frame.

## Methods

NewYork-Presbyterian/Weill Cornell Medical Center (NYP/WCM) is a large nonprofit academic medical center located in New York City. As of May 8, 2020, NYP/WCM has admitted a total of 1443 COVID-19 patients. At our institution, inpatient services are provided at NYP, while outpatient services are provided predominately at WCM facilities; both institutions share the same providers.

### Data Source

We used a customized version of the Press Ganey Outpatient Medical Practice Survey to evaluate patient satisfaction following clinic encounters at WCM from April 1, 2019, to March 31, 2020. The Press Ganey survey is used by more than 26,000 health care organizations, including over 60% of all US hospitals. It is the most commonly used, validated tool for assessing patient satisfaction in the outpatient setting [11]. The data contained deidentified patient-level data with the following variables: date of survey, visit type, patient age, gender, first visit (yes vs no), and Press Ganey satisfaction score (0%-100%). Visit type in Press Ganey is the same as the outpatient visit category coded in the outpatient electronic health record (Epic). There were over 200 visit types and 40 specialties represented. All video visits were synchronous video-based provider-patient visits scheduled and accessed through the outpatient enterprise electronic health record. WCM has been reimbursed with telemedicine payment parity since 2018.

The WCM Press Ganey Medical Practice Survey contains 31 items assessed on a 5-point Likert scale (ie, very poor, poor, fair, good, very good) to evaluate seven domains of patient care: Background Questions (3 items), Access (8 items), Moving Through Your Visit (4 items), Nurse/Assistant (3 items), Care Provider (6 items), Personal Issues (4 items), and Overall Assessment (3 items). WCM surveyed patients with 19 items from the standardized Press Ganey Outpatient Medical Practice Survey; the remaining 12 were by WCM from the Press Ganey item bank. The same survey instrument was used across all specialties and providers without variation for video vs in-person visits. Press Ganey sent the survey instrument 2-3 days after completion of the outpatient visit or video visit. Press Ganey then reported deidentified satisfaction scores to WCM without linkage to the patient’s electronic health record to maintain confidentiality.

### Study Population

We performed a retrospective study of patients aged 18 years and older. In order to adjust for the COVID-19 pandemic, we categorized visits after March 3, 2020, as the *COVID-19 period*. This timing corresponds with a WCM mandate to shift the majority of outpatient care from in-person to video visits.

**Figure 1:**
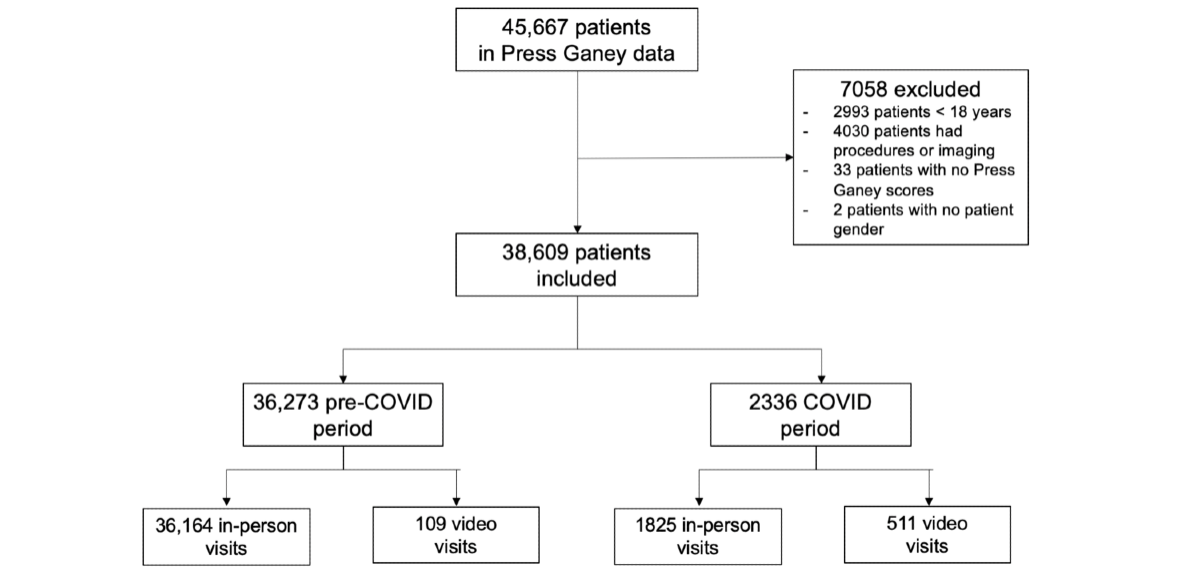
CONSORT (Consolidated Standards of Reporting Trials) diagram detailing the inclusion and exclusion criteria for our study.

Our total data included 45,667 outpatient visits across 210 visit categories with 2670 (5.8%) outpatients visits during the COVID-19 period. We defined the study group as consisting of video visits, which were identified if the visit type contained the words “Video Visit.” A total of 7058 outpatient visits were excluded: 4030 outpatient visits involving procedures (eg, surgery, venipuncture) or imaging, 2993 visits comprising pediatric patients <18 years of age, 33 visits that did not have Press Ganey scores, and 2 visits that did not include patient gender. The comparison group consisted of in-person outpatient visits. After applying our exclusion criteria, our final WCM Press Ganey data included 38,609 visits across 88 visit categories; of these, 620 (1.6%) video visits constituted the study population (Figure 1).

### Statistical Analysis

Independent variables were compared by pre–COVID-19 vs COVID-19 period using the paired *t* test and the chi-squared test. Hypothesis testing was conducted using the nonparametric Wilcoxon-Mann-Whitney test, comparing Press Ganey satisfaction scores between in-person and video visits across the study period. Additionally, we compared in-person and video visits in the pre–COVID-19 period to in-person and video visits during the COVID-19 period, respectively.

The dependent variable in our study was the Press Ganey patient satisfaction score. We fit a multivariable linear regression model with the following covariables: video visit (vs in-person), gender, age (18-25, 26-39, 40-59, 60-79, and 80 years [reference]), COVID-19 period (yes vs no), and new vs established visit.

Significance was established at *P*<.05. Statistical analysis was performed in R (version 4.0.0, The R Project for Statistical Computing) and STATA 14.0 (StataCorp LLC). The study was approved by the WCM Institutional Review Board and patient consent was not required for this study.

## Results

NYP/WCM experienced an 8729% increase in video visit use during the COVID-19 period compared to the pre–COVID-19 period.

The mean age of the total study population was 58.8 years (SD 16.5 years). Patients were slightly older in the pre–COVID-19 period than COVID-19 period (59.85 years vs 59.08 years; *P*=.03) (Table 1). There were no differences in gender or other visit characteristics between the two time periods.

In the pre–COVID-19 period, very few outpatient visits types were video visits (0.3%). During the COVID-19 period, video visits comprised 21.9% of outpatient visits. The proportion of all in-person visit types decreased except for postoperative visits. The visit type “follow-up” showed the greatest decline in share of outpatient visits, decreasing from 50.0% to 36.5% over the past year. Internal medicine outpatient visits constituted the greatest proportion of visits in both time periods.

**Table 1:** Baseline characteristics of clinic visits before and after COVID-19.

Characteristic				Pre–COVID-19 (April 1, 2019 to March 3, 2020) (n=36,273)		COVID-19 (March 4-31, 2020) (n=2336)		*P* value
**Press Ganey score (%), mean (SD)**								
	All visits			92.47 (11.25)		93.43 (10.51)		<.001
	Telemedicine			95.01 (8.65)		94.87 (10.22)		.31
	In-person			92.46 (11.26)		93.02 (10.56)		.004
**Age (years)**								
	Median (IQR)			63 (48-72)		62 (47-72)		—^a^
	Mean (SD)			59.85 (16.49)		59.08 (16.15)		.03
	**Category, n (%)**						<.001	
		18-25	853 (2.35)		46 (1.97)			
		26-39	4827 (13.31)		315 (13.48)			
		40-59	9526 (26.26)		713 (30.52)			
		60-79	17,911 (49.38)		1089 (46.62)			
		≥80	3156 (8.70)		173 (7.41)			
**Sex, n (%)**							.29	
	Male			14,444 (39.82)		904 (38.70)		
	Female			21,829 (60.18)		1432 (61.30)		
**Type of visit, n (%)**							<.001	
	Video			109 (0.30)		511 (21.88)		
	Follow-up			18,131 (49.98)		852 (36.47)		
	New patient			7606 (20.97)		393 (16.82)		
	Established well visit			3204 (8.83)		157 (6.72)		
	Consultation			1224 (3.37)		74 (3.17)		
	New well visit			1215 (3.35)		77 (3.30)		
	Follow-up (complex)			1132 (3.12)		55 (2.35)		
	Physical			772 (2.13)		44 (1.88)		
	Post-op			390 (1.08)		30 (1.28)		
	Other			2490 (6.86)		143 (6.12)		
**Visit type, n (%)**							.15	
	New			9816 (27.06)		665 (28.47)		
	Existing			26,457 (72.94)		1671 (71.53)		
**Specialty**							<.001	
	Internal medicine			7427 (20.47)		626 (26.80)		
	Obstetrics/gynecology			3287 (9.06)		206 (8.82)		
	Cardiology			3015 (8.31)		163 (7.00)		
	Ophthalmology			2863 (7.89)		144 (6.16)		
	Otolaryngology			2611 (7.20)		133 (5.69)		
	Hematology/oncology			1934 (5.33)		119 (5.09)		
	Dermatology			1646 (4.54)		84 (3.60)		
	Other			13,491 (37.19)		861 (36.86)		

^a^Not applicable.

**Figure 2:**
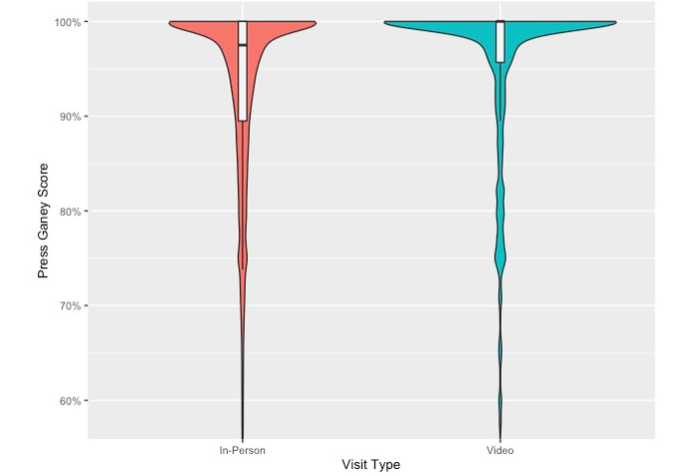
Violin and box-and-whiskers plot depicting the unadjusted distribution of in-person vs video visits.

Press Ganey patient satisfaction scores were significantly higher in the COVID-19 period when compared to the pre–COVID-19 period (93.4% vs 92.5%, *P*<.001). Notably, across the study period, patient satisfaction with video was significantly higher than in-person visits (94.9% vs 92.5%, *P*<.001) (Figure 2); this association was consistent during the pre–COVID-19 (95.0% vs 92.5%, *P*<.001) and COVID-19 periods (94.9% vs 93.0%, *P*<.001). While Press Ganey scores with video visits did not change across time periods (95.0% vs 94.9%, *P*=.31), the scores for in-person visits increased in the COVID-19 period (92.5% vs 93.0%, *P*=.004).

Overall, all of our covariables were statistically significant (Table 2). In adjusted analyses, video visits (parameter estimate [PE] 2.18; 95% CI 1.20-3.16), the COVID-19 period (PE 0.55; 95% CI 0.04-1.06), and the age category 60-79 years (PE 0.70; 95% CI 0.29-1.11) were associated with higher Press Ganey scores. Female gender (PE –0.73; 95% CI –0.96 to –0.50) and new visit type (PE –0.75; 95% CI –1.00 to –0.49) were associated with lower Press Ganey satisfaction scores.

**Table 2:** Multivariable linear regression for variables predicting Press Ganey scores.

Variable		Press Ganey score		*P* value
		Parameter estimate	95% CI	
Telemedicine		2.18	1.20 to 3.16	<.001
Female		–0.73	­–0.96 to –0.50	<.001
**Age (years) (reference: ≥80 years)**				
	18-25	–2.05	–2.88 to –1.22	<.001
	26-39	–1.95	–2.45 to –1.46	<.001
	40-59	–0.66	–1.10 to –0.22	.003
	60-79	0.70	0.29 to 1.11	.001
COVID-19 period		0.55	0.04 to 1.06	.04
New visit		–0.75	–1.00 to –0.49	<.001

## Discussion

Traditionally, health care encounters between a provider and a patient have occurred face to face in a physical location. Over the past 20 years, the internet and technology have made it possible for health care to be delivered digitally, providing new avenues for medicine to improve the value of care. Bridging gaps in time and distance, video visits have enabled providers to remotely care for patients with acute stroke [5], requiring intensive care [12], and located in rural areas or prison [13]. Half the hospitals in the United States report providing telehealth-based services [14]. Two years ago, the concept of a “medical virtualist” was created to describe a new specialty in which physicians primarily deliver care digitally [15]. The strengths of telemedicine have made it an indispensable tool in the clinical response to the COVID-19 pandemic. With the removal of financial disincentives and privacy barriers that limited widespread adoption, use of telemedicine has grown substantially in the United States during the COVID-19 pandemic. The 8729% increase in video visit utilization at our academic medical center is akin to the 4345% increase at New York University Langone Health [16] and the 4000% increase at Partners Healthcare [17].

Telemedicine is a new, and potentially disruptive, innovation and must be shown to be safe, effective, patient-centered, timely, efficient, and equitable [18]. Clinical consultations conducted through video visits are associated with high patient satisfaction [19,20] and lower costs [21-23] without a difference in clinical outcomes [13,24-27] compared to in-person consultations. However, most of these conclusions are based on evidence from small studies focused on remote telemedicine in sparsely populated locations or highly specific patient populations (eg, stroke care, rural ICUs, prisons) [5,12,13] not relevant to the care delivered during the COVID-19 pandemic [28]. Of all studies evaluating quality in telemedicine, four systematic reviews demonstrate there is limited published evidence to evaluate patient satisfaction as a metric for comparison to in-person visits [29-32]. This is problematic because patient satisfaction has been cited as the most important factor in the success of telemedicine initiatives [33]. Patient satisfaction as a measure of quality of care is a valid outcome [34] and a key component to value-based care [35]. Patient satisfaction is also associated with treatment plan adherence [36], reduced surgical readmissions [37], and patient retention [38].

Using the most current Press Ganey satisfaction scores, we found that video visits were associated with greater patient satisfaction when compared to in-person visits, which was not what we initially hypothesized. However, our results do not justify the use of telemedicine in lieu of in-person visits if both were equally accessible given the limitations outlined below. Furthermore, we observed that overall patient satisfaction is higher in the COVID-19 period, and that younger age, female gender, and new visit type were associated with lower patient satisfaction. To our knowledge, this is the largest study of patient satisfaction comparing video to in-person visits. The results of our study have particular relevance due to the unprecedented public health crisis that has necessitated the widespread adoption of video visits for patient safety and practicality.

Our study must be interpreted in the context of the study design. First, this is a retrospective study that prevents us from establishing causality between video visits and increased patient satisfaction. However, our data captures a rapid transition of outpatient care to video visits associated with the COVID-19 pandemic, and our analysis was adjusted for time. Second, our deidentified data did not capture patient-level variables that may influence Press Ganey scores, such as race, income, education, comorbidities, and other characteristics as they were not reported to WCM by Press Ganey to maintain patient confidentiality. Third, the same Press Ganey survey items were used for both video and in-person visits even though Press Ganey developed a new telemedicine version of the Medical Practice Survey in 2018 that may better characterize unique aspects pertinent to video visits [39]. Fourth, we were unable to assess for nonresponder bias; respondents to Press Ganey surveys have been shown to be more satisfied and more willing to respond than nonrespondents [40]. Fifth, what constitutes a clinically significant difference in patient satisfaction using Press Ganey survey scores is not well established. However, that said, there is mounting evidence in recent years that Press Ganey patient satisfaction score increases of 1%-10% can be viewed as clinically relevant [41-44].

In conclusion, we demonstrate that patient satisfaction with video visits compared favorably with in-person visits over the past year and during the COVID-19 pandemic. Our findings support the use of video visits as a viable alternative to traditional in-person visits. The New York City experience may offer insights into the future use of video visits as a new paradigm for health care delivery generally and in times of public health crisis.

## Abbreviations

ICU: intensive care unit
NYP/WCM: NewYork-Presbyterian/Weill Cornell Medical Center
PE: parameter estimate
